# The acute and sub-chronic effects of cocoa flavanols on mood, cognitive and cardiovascular health in young healthy adults: a randomized, controlled trial

**DOI:** 10.3389/fphar.2015.00093

**Published:** 2015-05-20

**Authors:** Laura A. Massee, Karin Ried, Matthew Pase, Nikolaj Travica, Jaesshanth Yoganathan, Andrew Scholey, Helen Macpherson, Greg Kennedy, Avni Sali, Andrew Pipingas

**Affiliations:** ^1^Centre for Human Psychopharmacology, Swinburne University of TechnologyMelbourne, VIC, Australia; ^2^National Institute of Integrative MedicineMelbourne, VIC, Australia; ^3^Department of Neurology, Boston University School of MedicineBoston, MA, USA

**Keywords:** cocoa, chocolate, flavanols, cognition, mental fatigue, cardiovascular, mood

## Abstract

Cocoa supplementation has been associated with benefits to cardiovascular health. However, cocoa's effects on cognition are less clear. A randomized, placebo-controlled, double-blind clinical trial (*n* = 40, age *M* = 24.13 years, *SD* = 4.47 years) was conducted to investigate the effects of both acute (same-day) and sub-chronic (daily for four-weeks) 250 mg cocoa supplementation on mood and mental fatigue, cognitive performance and cardiovascular functioning in young, healthy adults. Assessment involved repeated 10-min cycles of the Cognitive Demand Battery (CDB) encompassing two serial subtraction tasks (Serial Threes and Sevens), a Rapid Visual Information Processing task, and a mental fatigue scale over the course of half an hour. The Swinburne University Computerized Cognitive Assessment Battery (SUCCAB) was also completed to evaluate cognition. Cardiovascular function included measuring both peripheral and central blood pressure and cerebral blood flow. At the acute time point, consumption of cocoa significantly improved self-reported mental fatigue and performance on the Serial Sevens task in cycle one of the CDB. No other significant effects were found. This trial was registered with the Australian and New Zealand Clinical Trial Registry (Trial ID: ACTRN12613000626763). Accessible via http://www.anzctr.org.au/TrialSearch.aspx?searchTxt=ACTRN12613000626763&ddlSearch=Registered.

## Introduction

In recent years, there has been increasing attention given to the potential cognitive enhancing benefits of natural interventions, specifically foods containing flavonoids, the largest subclass of polyphenol antioxidants. Flavonoids and flavanols are abundant in the human diet, with popular dietary sources including tea, fruits, berries and cocoa (Bravo, 1998). The consumption of flavanol rich foods has been shown to result in neurobiological effects, improving aspects of learning, memory, and overall cognitive performance (Letenneur et al., 2007; Macready et al., 2009; Nurk et al., 2009; Williams and Spencer, 2012). In particular, cocoa and cocoa-containing products comprise many different forms of beneficial flavonoids (Gu et al., 2004) and as such, there has been increasing research aimed at uncovering the cognitive enhancing potential of high-flavanol cocoa.

It is important to differentiate between the terms cacao, cocoa and chocolate. The raw seeds obtained from the *Theobroma cacao* tree are referred to as cacao, however once these seeds are processed by way of grinding or roasting the name changes to cocoa (Latif, 2013). Chocolate confectionary involves further processing and the addition of multiple other ingredients including sugar and fat, resulting in a solid edible product (Cooper et al., 2008). The seeds of the *T. cacao* tree are rich in a subclass of polyphenol antioxidants known as flavonoids, specifically catechin and epicatechin, as well as polymeric units which comprise both catechin and epicatechin subunits, which are collectively referred to as proanthocyanidins (or procyanidins) (Steinberg et al., 2003; Nehlig, 2013). Cacao seeds have been used medicinally for centuries. Writings of the Mesoamerican civilizations describe the use of a beverage made from cacao seeds for the treatment of many ailments (Katz et al., 2011; Lippi, 2013). Cacao seeds were brought to Europe by Spanish explorers (Lippi, 2009), where cocoa was recognized for its health benefits until the 20th century when modern day manufacturing enabled the production of chocolate confectionary to begin. Since then, chocolate has been perceived as lacking nutritional value due to additional components such as fats and sugar, however, if certain controlled processing standards are implemented, substantial amounts of antioxidants from the original cacao seed can be retained in some modern day chocolates (Steinberg et al., 2003).

The human digestive system is able to readily absorb the epicatechin component of flavanol-rich cocoa, with blood plasma concentrations peaking at 2–3 h following consumption (Nehlig, 2013). Epicatechin has been shown to cross the blood brain barrier in animal studies (Abd El Mohsen et al., 2002), suggesting that flavonoids from the cacao bean have the ability to act directly on the brain, which could potentially lead to cognitive enhancement (Nehlig, 2013). Additional evidence from animal studies on flavanols in fruits such as grapes and berries found that flavanol consumption boosts memory and learning (Shukitt-Hale et al., 2006, 2008; Joseph et al., 2007) by acting directly on numerous receptors, kinases and transcription factors (Spencer, 2007, 2009). As such, flavanols in cocoa may also have the potential to act directly on the human brain and improve aspects of cognitive performance through direct enhancement of memory systems, just as the flavanols in fruit have been shown to do.

Cocoa may not only exert direct effects on the brain, but could potentially act indirectly through effects to other bodily systems. It has long been known that cocoa has beneficial effects on the cardiovascular system, with initial evidence coming from observational studies on the Kuna Indians, residing off the coast of Panama. The Kuna are one of few remaining tribes who are protected from vascular ailments, such as arterial hypertension (Corti et al., 2009). It has been suggested that this is due to the prominent role cocoa plays in the Kuna diet (Galleano et al., 2009; Hollenberg et al., 2009), where they consume the equivalent of five cups of liquid cocoa, or approximately 900 mg cocoa flavanols, per day (Pucciarelli, 2013). Research demonstrated that when members of the Kuna relocated to Panama, changing their culture and diet (in particular a reduced dietary intake of flavanol-rich cocoa), significant increases in blood pressure were subsequently observed (Galleano et al., 2009; Fraga et al., 2011). Clinical investigations have revealed that supplementation with flavanol-rich cocoa reliably improves a variety of cardiovascular variables, including peripheral blood pressure in pre-hypertensive and hypertensive patients (Taubert et al., 2007; Hooper et al., 2008; Ried et al., 2010), as well as in normotensive populations (Ried et al., 2012). Changes to cerebral blood flow (CBF) (Fisher et al., 2006; Sorond et al., 2008) and flow-mediated dilation (Faridi et al., 2008; Grassi et al., 2008; Monahan et al., 2011; Njike et al., 2011) have been observed following cocoa supplementation. Given the strong links between vascular health and cognitive function (Dinges, 2006), it is possible that cocoa may improve cognitive performance indirectly through improvements to CBF and vascular health.

Randomized controlled trials have investigated the possibility that cognitive function may be improved following cocoa consumption. A short term neuroimaging investigation in young, healthy, female participants revealed that acute administration of a high flavanol cocoa beverage (172 mg/day for 5 days) enhanced the Blood Oxygen Level Dependent (BOLD) signal following completion of a cognitive switching task (Francis et al., 2006). Also using an acute intervention, Field et al. (2011) found that 2 h after cocoa-flavanol supplementation, young participants showed enhanced performance on the accuracy component of a spatial working memory task, as well as performance improvements on a choice reaction time task. Cognitive performance has been enhanced following cocoa beverage supplementation in elderly people with mild cognitive impairment (Desideri et al., 2012). In contrast, some studies have found no such cognitive effects, for instance, Pase et al. (2013) found no changes to cognitive performance acutely or sub-chronically for either a 250 or 500 mg cocoa flavanol dose in healthy participants (40–65 years). Crews et al. (2008) found no cognitive effects in a healthy, older sample (>60 years) following a 6 week intervention with a cocoa beverage (397.30 mg flavanols) and bar (357.41 mg flavanols). More recently, however, Scholey et al. (2010) found acute improvements in cognitive performance following a 520 mg cocoa flavanol dose, in response to a high level of cognitive demand. In this same study, cocoa flavanols also reduced self-reported mental fatigue. Both Pase et al. (2013) and Crews et al. (2008) did not implement a mentally demanding task, suggesting that cocoa may only improve cognitive performance in healthy populations when participants are subjected to sustained effortful processing.

The aim of the current randomized, controlled, double-blind clinical trial was to assess both the acute (2 h post dose), and sub-chronic (30 day post dose) effects of cocoa supplementation on mood and mental fatigue, cognitive performance and cardiovascular function in a young, healthy sample. Unlike previous clinical trials investigating cocoa and cognition, the current trial provided participants with either a visually identical cocoa or true placebo tablet without flavanols. By providing the supplement in tablet form, participant expectation is removed as they cannot visually decipher which treatment is the placebo and which is active, compared to other trials which used dark and white chocolate supplements (Field et al., 2011). Additionally, by making use of a tablet, other potentially active ingredients like sugars and fats can be excluded in order to make sure that any resulting effects can reliably be attributed to cocoa flavanols, and not to any of the other components found in commercial chocolate or other styles of intervention such as chocolate drinks (Francis et al., 2006; Crews et al., 2008; Scholey et al., 2010; Desideri et al., 2012; Pase et al., 2013). Participants were randomly allocated to receive tablets containing either 250 or 0 mg (placebo) cocoa-flavanols. It was hypothesized that, compared to placebo, cocoa would improve cognitive performance and mood.

## Methods

### Participants

**Figure 1:**
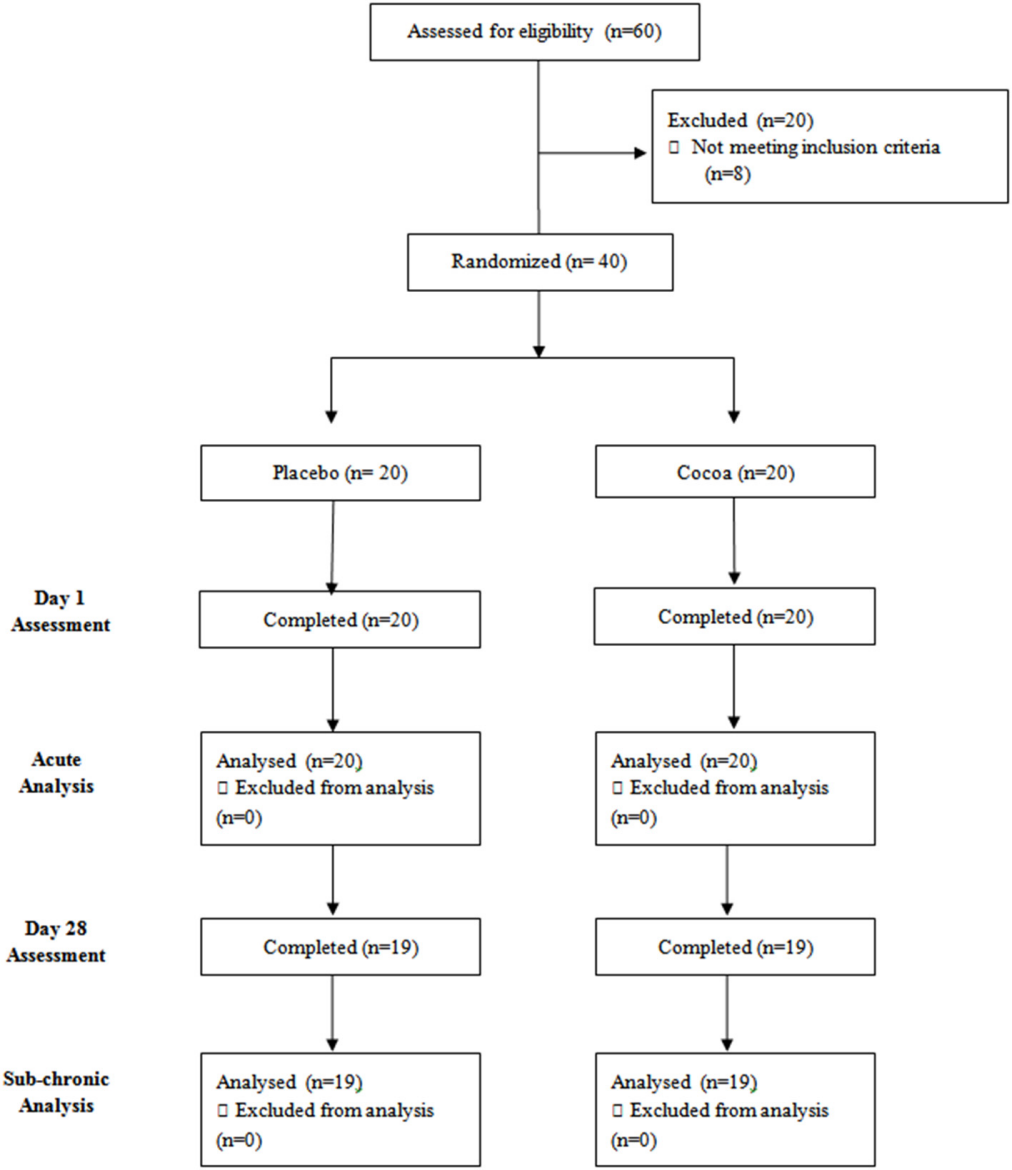
**Study flow diagram**.

Young, healthy participants aged 18–40 years (*M* = 24.13, *SD* = 4.47), residing in Melbourne, Australia, were recruited through magazine and social media advertisements, phone calls, and emails. Potential participants expressing interest in the study were screened for the following inclusion criteria: not currently suffering medically diagnosed cardiovascular or cognitive impairment, bleeding disorders, or gastrointestinal disorders; no clinically significant pulmonary, cardiovascular, psychiatric, or neurological conditions in the past 12 months; not taking any illicit drugs, cognitive enhancing medication, or herbal supplements; not pregnant or lactating; not color blind; not taking antidepressants, antipsychotics, anxiolytics, or anticoagulants; hold a good working knowledge of English language. Of the 40 recruited participants, 38 returned for sub-chronic assessment (see Figure 1).

### Design

The current trial investigated the effects of cocoa supplementation on cognitive and cardiovascular functioning using a randomized, placebo-controlled, double-blind, parallel design over a four-week period. Participants were randomly allocated to receive one of two daily treatments:

1. Active cocoa tablet (3058 mg *T. cacao* seed extract standardized to contain 250 mg catechin polyphenols and 5.56 mg caffeine).
2. Placebo tablet (Identical in appearance, size, texture and color to cocoa tablet, containing inert cellulose powder).

The active and matching placebo supplements used in this clinical trial were provided by Swisse Wellness Pty. Ltd. (Melbourne, Australia). Participants were required to take one active (or matching placebo) tablet daily for 4 weeks. The tablets were presented in visually identical bottles, differing only in the labels, which depicted the participant ID number as well as a code relating to the relevant group allocation. The placebo tablets were identical to the active tablets, and blinding success of participants was evaluated at the conclusion of the study using a questionnaire. Participants were randomly assigned to receive either active or placebo tablets using a computer generated permuted block randomization schedule. Randomization was performed by an independent statistical consultant that had no involvement in the trial, and supplement bottles were labeled according to the randomization schedule. The blinding code was only revealed after analysis of the main study outcomes.

### Outcome measures

The present study investigated the effect of cocoa supplementation on mood, cognitive and cardiovascular function between baseline and acute (same day, 2 h after ingestion of trial tablets), and baseline and sub-chronic (daily for 4 weeks) testing sessions. The primary outcomes were cognitive performance, measured using the Swinburne University Computerized Cognitive Assessment Battery (SUCCAB) (Pipingas et al., 2010), mood, mental fatigue and stress, using the Cognitive Demand Battery (CDB). Secondary outcomes were cardiovascular markers, including blood pressure and cerebral blood flow.

### Cognitive assessment

#### Swinburne university computerized cognitive assessment battery (SUCCAB)

Participants completed eight computer-based tasks from the SUCCAB to assess various aspects of cognitive performance (Pipingas et al., 2010). A 4-button response box was used to complete the tasks, with each button representing a color (red, blue, green or yellow), “yes” or “no,” or the spatial location of objects on the screen (top, bottom, left or right).

Instructions were presented at the beginning of each of the tasks, which were read aloud to participants by the experimenter. All tasks except the delayed recall task were preceded by a practice trial where participants could experience an example of the task and ask any questions. Each of the eight tasks was scored by calculating the average reaction time and per cent accuracy, and are described below in further detail.

(1) Simple reaction time: A single white square appeared in the center of the computer screen 30 times at randomized intervals. The “yes” button was required to be pressed as soon as the square stimulus was visible to participants.
(2) Choice reaction time: Either a blue triangle or red square appeared in the center of the computer screen with the order and timing of the stimuli being randomized. Responses were recorded by the pressing of either the blue or red buttons corresponding with the stimuli's color. This task assessed participants' visual perception decision time.
(3) Immediate recognition: Forty successive images were presented in the center of the computer screen. After the viewing period, participants were required to respond a second series of successive images with either “yes” or “no” buttons, signaling whether or not they had just seen a given image during the earlier viewing period. Half of these images in the second series were from the original 40, and half were novel stimuli. This task was a non-verbal assessment of recognition memory.
(4) Congruent Stroop color word: Randomized alternating trials of stimulus words (red, yellow, blue, or green) in congruent colors appeared on the computer screen (I.E. the word blue would be presented in blue ink). To respond, the color button that corresponded to the color of the word, not the physical presentation of the word itself needed to be pressed. This task assessed participants' executive functioning and inhibition, as they had to ignore the reflex of reading the word and focus instead on the color it was presented in.
(5) Incongruent Stroop color word: Conducted in the same way as congruent Stroop color word, except that the stimulus words (red, yellow, blue, or green) appeared in incongruent colors on the computer screen. (I.E. the word blue could be presented in red, green or yellow ink, but not blue ink). Participants' skills in executive functioning and inhibition are more vigorously tested in these incongruent trials.
(6) Spatial working memory: A 4 × 4 white grid was displayed against a black background, with six of the grid positions filled with solid white squares. The aim was to remember where these squares were located during a brief viewing period. The grid then became blank, and a series of four white squares were presented one at a time in varying grid locations. Participants responded with either the “yes” or “no” buttons indicating if the new squares were presented in the same locations as the original set. This task assessed participants' ability to hold spatial information in working memory.
(7) Contextual memory: A series of 20 everyday pictures that were positioned at either the top, bottom, left, or right of the computer screen were shown against a black background. After the viewing period, the same images were randomly presented in the center of the computer screen. Participants responded using either the top, bottom, left, or right buttons to indicate the image's original location on the screen. This task assessed participants' episodic memory capabilities, as they needed to recall the spatial location of the original stimuli.
(8) Delayed recognition: The immediate recognition task described earlier was repeated at the end of the testing battery without a practice trial. Participants were shown the remaining original 20 images and another 20 novel images, and were again required to respond “yes” or “no” as to whether or not they had viewed them earlier. As in the immediate recognition task, this also assessed recognition memory.

#### Cognitive demand battery (CDB)

**Figure 2:**
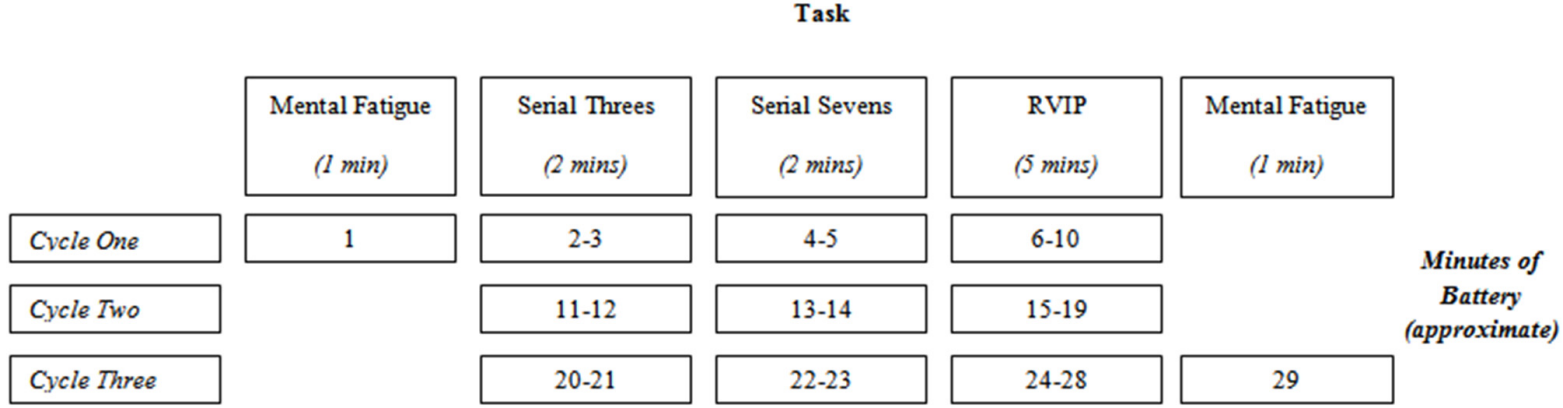
**Presentation order of the three CDB cycles**. RVIP, Rapid Visual Information Processing.

The CDB is used to evaluate cognitive function by placing participants under mental load. Moreover, this battery is designed to assess mental fatigue (and mood) resulting from the ongoing mental load. This battery involves the completion of two serial subtraction tasks (serial threes and serial sevens), the Bakan Rapid Visual Information Processing Task (RVIP) and a mental fatigue visual analog scale (Kennedy and Scholey, 2004). Research has shown that while completing this battery, participants' task performance gradually declines whilst they report feeling more mentally fatigued. Many studies looking at the cognitive effects of various dietary interventions have shown that this decline can be somewhat mitigated by supplementation. Figure 2 displays the structure of the CDB. There are three main components to the CDB (Serial Threes, Sevens and RVIP), which are both preceded and followed by a mental fatigue assessment.

1. Mental fatigue scales: Initially, participants are allocated 1 min to rate their current state of mental fatigue and stress, by marking a point on a computerized 100 mm Visual Analog Scale (VAS), with the left hand end of the scale labeled “not at all,” and the right hand end of the scale labeled “very much so.”
2. Serial Threes subtraction task: Participants are presented with a given number ranging between 800 and 999, and are required to use the number keys on the keyboard to count backwards by threes from this number as quickly and as accurately as possible. The original starting number is cleared from the screen upon entry of participants' first response. Two minutes are allocated for this task and participants are scored based on the number of correct responses, errors and speed of response.
3. Serial Sevens subtraction task: This task is identical in format to the Serial Threes subtraction task, however it required participants to count backwards by sevens. Two minutes were also allocated for this task.
4. Rapid Visual Information Processing Task (RVIP): Participants are required to observe a continuous presentation of single digits for targets of any three consecutive odd or even digits. One hundred digits were presented each minute and participants responded to the detection of targets by pressing the spacebar as quickly as possible. This task runs for 5 min and is scored based on the number of correctly detected targets, number of errors and number of false alarms.
5. Mental fatigue scales: Participants were again required to rate their current subjective mental fatigue and stress on the same 100 mm VAS used at the beginning of the CDB.

The duration for one cycle of the CDB is 10 min. Participants in the present study completed three cycles, resulting in a total duration of 30 min.

### Cardiovascular measures

#### Blood pressure

Blood pressure was assessed in a quiet, dedicated university laboratory following a five-minute rest period completed by participants in the supine position on an examination bed. The non-invasive SphygmoCor XCEL (AtCor Medical, Sydney, Australia) device and software was used to attain participants' peripheral and central aortic blood pressures, by applying the cuff to participants' right arm, over the brachial artery. The SphygmoCor XCEL takes three measurements of brachial blood pressure for each participant, each separated by a thirty second delay. The first measurement is discarded, while the second and third are averaged to provide a more accurate final reading. The SphygmoCor XCEL provides a valid estimation of central aortic blood pressure using the brachial cuff volume displacement waveform method (Butlin et al., 2012).

#### Cerebral blood flow

Also using a dedicated university laboratory, a non-invasive transcranial Doppler (Compumedics DWL, CA, USA) with a 4-MHz probe was used to record cerebral blood flow velocity in the common carotid artery (CCA). Experimenters recorded the flow through the CCA for approximately 2 min in order to calculate the resting mean CCA blood flow velocity in centimeters per second.

### Procedure

Participants completed a total of three testing sessions; baseline and acute assessment which took place on the same day (2–3.5 h after tablet ingestion), and sub-chronic assessment which was completed four-weeks after the initial testing date. Participants consumed their allocated treatment daily for 30 days. Participants were instructed to abstain from caffeinated products from the night prior to each testing session, as well as abstaining from consuming any food or drink with the exception of water for the duration of each of the testing days. The current trial implemented procedures, which were conducted in accordance with the Declaration of Helsinki (as revised in 2004). The Swinburne University Human Research Ethics Committee approved the trial, and written informed consent was obtained from all participants prior to the commencement of testing.

**Figure 3:**
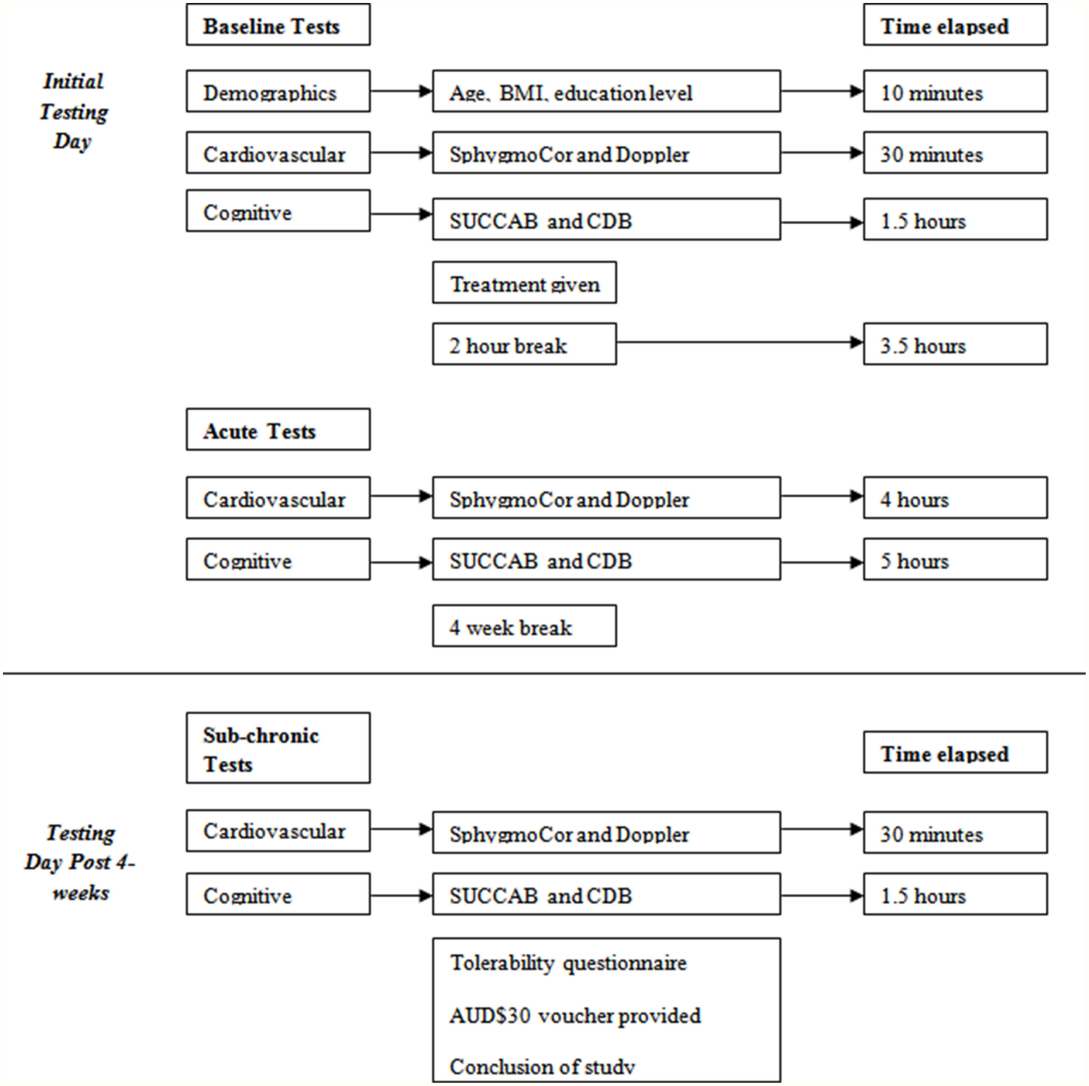
**Testing day structure**. BMI, Body Mass Index; SUCCAB, Swinburne University Computerized Cognitive Assessment Battery; CDB, Cognitive Demand Battery.

The first testing day included both baseline and acute assessment and ran for approximately 5 h, while the follow-up testing day involved sub-chronic assessment, which required approximately one and a half hours. All three testing sessions are outlined in Figure 3.

At the completion of the acute session, an appointment time for the post four-week testing session was scheduled, planned for the same time of day as the first testing session. Additionally, participants were instructed to take their final supplement tablet the day prior to the scheduled final testing session.

The follow up sub-chronic testing session was conducted exactly 4 weeks after the initial session. The session was conducted in a way synonymous to the baseline session. Participants were reminded to not consume any supplement tablets on the day of sub-chronic testing. This way, experimenters could be certain that they were measuring the sub-chronic, and not acute effects of cocoa. At the completion of the final testing session participants completed a questionnaire that evaluated their tolerability to the supplement over the four-week period, as well as the blinding success of group allocation. Participants were then reimbursed for their time and travel expenses with a $30 voucher.

#### Sample size calculations

A power-analysis was performed based on effect sizes obtained from Scholey et al. (2010)'s study. For mental fatigue, Scholey et al. (2010) reported effect sizes ranging from 0.33 to 0.42. To be 95% confident in finding a significant effect (*d* = 0.33) of cocoa supplementation on mental fatigue, a sample size of 26 participants (13 per group) was deemed sufficient. For cognitive performance Scholey et al. (2010) reported effect sizes ranging from 0.30 to 0.53. For this measure, a sample size of 32 participants (16 per group) was considered sufficient to be 95% confident in detecting a significant effect (*d* = 0.30). It was decided that a sample of 40 participants (20 per group) would allow for a 20% attrition rate, while also being powerful enough to have 95% confidence in detecting significant effects.

#### Statistical analysis

SPSS version 21 (IBM, New York, NY, USA) was used to compile and analyse all data. The data was first screened for any potential outliers, which were subsequently removed. Analyses of co-variance (ANCOVAs) were used to examine potentially significant differences between groups at the both the two-hour acute, and four-week sub-chronic time points, while taking baseline measures into account as a covariate. Additionally, *post hoc* independent samples *t*-tests were used to compare the characteristics of the treatment groups at baseline, while *post hoc* paired samples *t*-tests were used to compare any significant differences found when comparing baseline to acute or to sub-chronic assessment time points. All results were considered significant at *p* < 0.05. All *p*-values are calculated two-tailed.

## Results

### Sample characteristics

Table 1 displays the means and standard deviations for the two groups across baseline demographic variables. ANCOVAs revealed no significant between-group differences at baseline. Also noteworthy is that the sample had normal blood pressure levels at baseline.

**Table 1:** **Baseline means and standard deviations across each treatment condition**.

	**Cocoa**		**Placebo**		**Overall**	
	***M***	***SD***	***M***	***SD***	***M***	***SD***
**DEMOGRAPHIC VARIABLES**						
**Gender (%)**						
Males	20		12.5		32.5	
**Highest completed education (%)**						
Secondary school	5		10		7.5	
Technical school	10		10		10	
Bachelor degree	80		75		77.5	
Postgraduate degree	5		5		5	
Age (years)	24.35	4.75	23.90	4.28	24.13	4.47
BMI	22.40	3.07	23.60	3.19	23	3.15
**CARDIOVASCULAR VARIABLES**						
CCA velocity, cm/s	21.60	6.33	21.43	7.14	21.52	6.65
Heart rate, bpm	68.10	11.46	76.00	15.36	72.05	13.96
Systolic BP, mmHg	116.50	10.55	121.70	11.77	119.10	11.34
Diastolic BP, mmHg	70.00	9.66	71.45	8.86	70.73	9.18
Central systolic BP, mmHg	102.00	10.38	106.10	9.84	104.05	10.20
Central diastolic BP, mmHg	70.15	10.13	73.80	10.92	71.98	10.56
Mean arterial pressure, mmHg	83.65	10.64	88.05	11.40	85.85	11.11
Pulse pressure, mmHg	32.30	7.61	32.30	6.07	32.30	6.79
Augmented pressure, mmHg	2.47	3.84	2.44	2.53	2.46	3.22
Augmentation index, %	6.95	13.23	8.00	7.84	7.46	10.81

*CCA, Common Carotid Artery; BP, Blood Pressure; mmHg, Millimeters of Mercury*.

### Acute analysis

#### SUCCAB

No significant between-group differences were found for accuracy or reaction time in any SUCCAB task at the acute time point when co-varying for baseline data (Table 2).

**Table 2:** **Acute effects of cocoa supplementation on cognition: SUCCAB task accuracy and reaction time**.

	**Cocoa**				**Placebo**					
	**Baseline (0 h)**		**Acute (2 h)**		**Baseline (0 h)**		**Acute (2 h)**		**ANCOVA**	
	***M***	***SD***	***M***	***SD***	***M***	***SD***	***M***	***SD***	***F***	***p***
**TASK ACCURACY (%)**										
Simple reaction time	99.75	1.12	95.83	6.24	96.95	5.69	96.87	5.25	1.66	0.21
Complex reaction time	85.21	11.75	75.26	14.86	77.45	12.63	77.05	13.39	0.95	0.34
Immediate recognition	79.65	10.94	84.56	7.79	73.50	11.92	80.67	9.89	0.33	0.57
Congruent stroop	97.82	2.67	96.32	7.22	99.23	1.46	98.08	1.84	0.96	0.96
Incongruent stroop	97.63	2.43	93.68	4.03	98.00	4.18	94.63	4.00	0.41	0.53
Spatial working memory	91.16	4.54	89.02	8.05	91.51	9.46	89.42	11.37	0.001	0.97
Contextual memory	81.75	14.07	82.50	12.72	87.89	10.18	86.11	11.06	0.12	0.73
Delayed recognition	76.44	9.34	70.50	12.32	68.28	10.30	73.25	9.40	4.34	0.05
**TASK REACTION TIME (ms)**										
Simple reaction time	240.63	25.93	243.27	32.15	242.32	35.42	261.12	47.16	2.26	0.14
Complex reaction time	371.75	43.56	385.75	48.97	385.64	58.21	383.17	43.48	0.06	0.06
Immediate recognition	804.51	67.94	787.21	59.87	938.81	94.51	855.75	101.34	0.42	0.52
Congruent stroop	567.68	82.53	577.40	79.36	622.79	83.60	600.89	94.51	1.11	1.11
Incongruent stroop	660.83	87.55	675.17	104.37	683.74	89.51	669.65	94.89	0.76	0.39
Spatial working memory	693.90	79.66	651.73	84.71	737.65	107.38	679.96	88.21	0.002	0.97
Contextual memory	816.08	97.93	782.29	84.25	817.95	122.32	837.41	109.11	3.56	0.07
Delayed recognition	982.17	100.70	847.36	83.13	1029.26	154.85	894.49	97.61	0.73	0.40

#### CDB

Prior to commencing the CDB, participants receiving cocoa supplementation reported feeling significantly less mentally fatigued at the beginning of the battery, compared to participants receiving placebo supplementation (Table 3).

**Table 3:** **Acute effects of cocoa supplementation on self-reported mental fatigue and stress, before and after the cognitive demand battery**.

	**Group**	**Baseline (0 h)**		**Acute (2 h)**		**ANCOVA**	
		***M***	***SD***	***M***	***SD***	***F***	***p***
**BEFORE CDB**							
Mental fatigue	Cocoa	49.40	16.22	49.00	22.33	12.21	0.004^*^
	Placebo	46.26	15.38	63.17	11.59		
Stress	Cocoa	22.70	14.43	31.30	14.62	0.50	0.50
	Placebo	29.94	14.89	38.94	17.17		
**AFTER CDB**							
Mental fatigue	Cocoa	64.00	17.82	62.05	17.48	0.23	0.63
	Placebo	70.32	16.04	66.89	15.70		
Stress	Cocoa	37.00	18.63	31.30	18.71	0.54	0.47
	Placebo	47.94	16.68	41.65	16.56		

*CDB, Cognitive Demand Battery*.

* *p < 0.05*.

**Figure 4:**
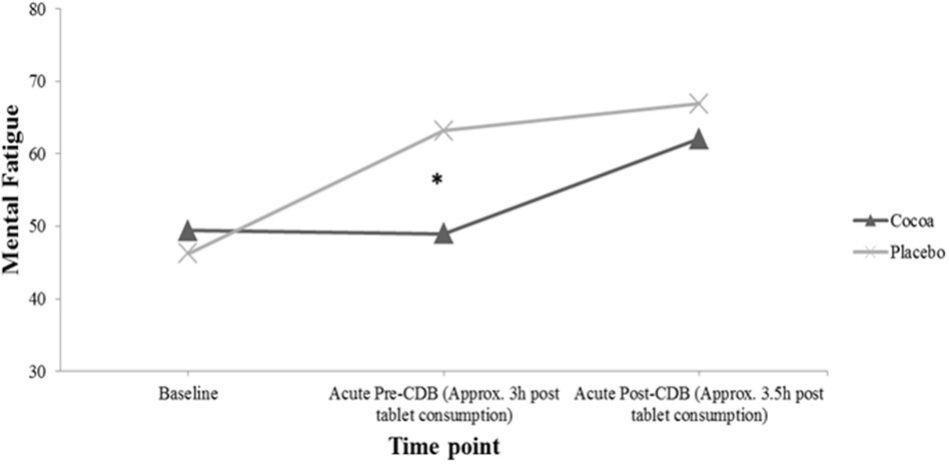
**Participants average response to the question “how mentally fatigued do you feel right now” both before, and after acute tablet consumption**. (For mental fatigue, responses to the question range from 1 = “not at all” to 100 = “very much so”). Significant differences compared to placebo are indicated (^*^p < 0.05). CDB, Cognitive Demand Battery.

Figure 4 displays participants responses to the question “How mentally fatigued do you feel right now,” which were found to be significantly different at the acute-pre CDB time point in the ANCOVA analysis presented in Table 3. *Post hoc* independent samples *t*-tests revealed that while the treatment groups were not significantly different at baseline [*t*_(37)_ = 0.62, *p* = 0.54], participants receiving cocoa reported being significantly less mentally fatigued (*M* = 49.00, *SD* = 22.33) than participants receiving placebo supplementation (*M* = 63.17, *SD* = 11.59) at the acute pre-CDB time point [*t*_(29.16)_ = −2.49, *p* = 0.02]. While the groups were not significantly different in the ANCOVA analysis at the acute post-CDB time point, Figure 4 shows that both groups did report being more mentally fatigued after completing the acute CDB compared to previous time points, with participants in the placebo group reporting feeling more mentally fatigued than participants receiving cocoa.

Significant between-group differences were found for the Serial Sevens task at the acute time point when co-varying for baseline, with participants receiving cocoa providing significantly more correct answers than those in receiving placebo. No significant between-group differences were observed during cycles two and three of the CDB (Table 4).

**Table 4:** **Acute effects of cocoa supplementation on cognition: CDB Cognitive Tasks**.

		**Cycle 1**						**Cycle 2**						**Cycle 3**					
***CDB Task***	**Group**	**Baseline (0 h)**		**Acute (2 h)**		**ANCOVA**		**Baseline (0 h)**		**Acute (2 h)**		**ANCOVA**		**Baseline (0 h)**		**Acute (2 h)**		**ANCOVA**	
		***M***	***SD***	***M***	***SD***	***F***	***p***	***M***	***SD***	***M***	***SD***	***F***	***p***	***M***	***SD***	***M***	***SD***	***F***	***p***
Serial threes correct (number)	Cocoa	24.30	12.08	37.25	15.25	0.97	0.97	31.60	12.63	37.80	15.80	0.61	0.44	33.00	13.07	39.75	16.02	0.02	0.88
	Placebo	20.58	9.73	33.37	16.59			25.74	14.27	34.00	16.47			29.35	15.64	36.88	15.31		
Serial threes speed (ms)	Cocoa	5602.35	3339.54	3277.55	1263.80	0.29	0.29	3820.55	1833.90	3524.00	1853.82	0.74	0.40	3794.05	1659.03	3225.25	1328.97	0.006	0.94
	Placebo	6053.37	2515.80	3855.37	1784.40			4368.89	1553.43	3668.32	1500.48			4147.53	1521.50	3521.06	1698.35		
Serial sevens correct (number)	Cocoa	14.90	10.94	21.55	11.20	5.93	0.02^*^	17.10	10.12	21.65	12.53	3.44	0.07	18.20	10.59	23.20	12.73	0.29	0.60
	Placebo	13.95	8.87	15.39	9.22			15.32	7.75	15.61	9.74			13.76	7.63	17.76	8.19		
Serial sevens speed (ms)	Cocoa	7728.10	4473.64	5623.75	2359.32	3.77	0.06	6816.70	3526.88	5828.75	3419.20	3.08	0.08	6730.60	3163.55	5512.30	2950.15	1.52	0.23
	Placebo	8155.42	3097.95	6837.65	2696.41			6628.63	1915.32	7414.67	3668.25			7235.47	2281.21	6636.65	2548.32		
RVIP accuracy (%)	Cocoa	44.35	23.55	51.87	27.72	0.03	0.86	49.82	24.17	52.81	23.75	0.26	0.61	43.21	29.54	51.84	22.88	0.55	0.46
	Placebo	33.29	14.98	42.24	16.87			40.00	16.60	41.97	15.36			38.08	18.26	44.56	16.09		
RVIP speed (ms)	Cocoa	453.74	57.30	483.53	69.05	0.30	0.59	475.03	56.92	482.17	50.63	0.11	0.74	478.97	42.83	473.30	49.53	1.48	0.23
	Placebo	472.70	53.90	479.82	66.06			494.19	47.92	499.30	65.14			493.75	53.52	500.00	55.12		

*RVIP, Rapid Visual Information Processing*.

* *p < 0.05*.

#### Cardiovascular measures

No significant effects were found for any of the cardiovascular measures at the acute time point when co-varying for baseline data (Table 5).

**Table 5:** **Acute effects of cocoa supplementation on cardiovascular measures**.

	**Cocoa**				**Placebo**				**ANCOVA**	
	**Baseline (0 h)**		**Acute (2 h)**		**Baseline (0 h)**		**Acute (2 h)**			
	***M***	***SD***	***M***	***SD***	***M***	***SD***	***M***	***SD***	***F***	***p***
**CARDIOVASCULAR MEASURES**										
CCA velocity, cm/s	21.60	6.33	20.39	6.76	21.43	7.14	23.64	5.71	3.19	0.09
Heart rate, bpm	68.10	11.46	64.05	12.36	76.00	15.36	70.05	14.06	0.02	0.89
Systolic BP, mmHg	116.50	10.55	119.10	9.04	121.70	11.77	120.65	8.24	0.47	0.50
Diastolic BP, mmHg	70.00	9.66	68.80	6.62	71.45	8.86	69.60	7.26	0.003	0.96
Central systolic BP, mmHg	102.00	10.38	103.35	8.73	106.10	9.84	104.45	6.68	0.42	0.42
Central diastolic BP, mmHg	70.15	10.13	69.85	7.06	73.80	10.92	70.70	7.96	0.53	0.47
Mean arterial pressure, mmHg	83.65	10.64	82.75	7.93	88.05	11.40	84.40	8.26	0.47	0.50
Pulse pressure, mmHg	32.30	7.61	33.50	4.54	32.30	6.07	33.85	6.56	0.06	0.81
Augmented pressure, mmHg	2.47	3.84	2.37	3.40	2.44	2.53	2.31	2.89	0.002	0.97
Augmentation index, %	6.95	13.23	6.74	10.06	8.00	7.84	7.38	9.29	0.001	0.98

*CCA, Common Carotid Artery; BP, Blood Pressure; mmHg, Millimeters of Mercury*.

### Sub-chronic analysis

#### SUCCAB

No significant between-group differences were found for accuracy or reaction time in any SUCCAB task at the sub-chronic time point when co-varying for baseline data (Table 6).

**Table 6:** **Sub-chronic effects of cocoa supplementation on cognition: SUCCAB task accuracy and reaction time**.

	**Cocoa**				**Placebo**				**ANCOVA**	
	**Baseline (0 h)**		**Sub-chronic (4 week)**		**Baseline (0 h)**		**Sub-chronic (4 week)**			
	***M***	***SD***	***M***	***SD***	***M***	***SD***	***M***	***SD***	***F***	***p***
**TASK ACCURACY (%)**										
Simple reaction time	99.74	1.15	97.50	3.93	96.79	5.80	96.76	5.85	1.03	0.32
Complex reaction time	84.43	11.53	80.53	11.77	76.32	11.88	75.28	12.30	0.68	0.42
Immediate recognition	78.89	10.72	79.84	13.70	72.98	12.01	78.49	11.84	0.34	0.56
Congruent stroop	97.84	2.74	96.84	3.21	99.19	1.49	98.55	1.92	0.61	0.22
Incongruent stroop	97.50	2.43	94.31	3.91	98.82	2.10	95.00	2.89	0.002	0.96
Spatial working memory	90.88	4.49	93.47	5.50	91.07	9.50	90.30	9.65	2.34	0.14
Contextual memory	80.79	13.78	87.50	12.98	87.50	10.33	88.61	8.19	0.02	0.90
Delayed recognition	75.90	9.27	74.17	19.34	68.28	10.30	72.01	18.04	0.81	0.38
**TASK REACTION TIME (ms)**										
Simple reaction time	240.46	26.63	240.13	40.91	242.16	36.50	254.62	46.68	0.79	0.38
Complex reaction time	374.66	42.71	363.89	31.16	385.64	58.21	367.62	40.93	0.10	0.76
Immediate recognition	809.05	66.87	807.15	85.12	939.34	97.07	843.12	79.44	0.56	0.46
Congruent stroop	570.80	83.57	594.43	92.39	625.60	84.91	614.38	85.43	1.59	0.44
Incongruent stroop	667.32	85.26	662.20	108.35	680.43	90.70	680.14	69.05	0.30	0.59
Spatial working memory	695.85	81.35	641.06	82.61	743.67	106.79	694.15	89.73	0.62	0.44
Contextual memory	820.07	98.93	778.84	99.84	822.32	124.33	793.79	95.15	0.25	0.62
Delayed recognition	983.40	103.31	849.41	108.80	1029.26	154.85	901.04	124.46	0.74	0.40

#### CDB

Looking at sub-chronic data after the third cycle of the CDB, participants receiving the placebo reported feeling significantly less stressed compared to those receiving cocoa (Table 7). *Post-hoc* independent samples *t*-tests revealed that the treatment groups were not significantly different at baseline [*t*_(34)_ = −1.83, *p* = 0.08] or sub-chronic [*t*_(34)_ = 0.54, *p* = 0.59] assessment. Further *post hoc* paired *t*-test analyses were subsequently completed to compare stress levels over time separately for each of the groups. Results revealed that while the participants receiving cocoa did not report any significant changes in subjective stress levels over time [*t*_(17)_ = −0.88, *p* = 0.39], participants receiving placebo supplementation did report significantly lower levels of stress after completing the CDB at sub-chronic assessment relative to baseline [*t*_(16)_ = 3.43, *p* = 0.003].

**Table 7:** **Sub-chronic effects of cocoa supplementation on self-reported mental fatigue and stress, before and after the cognitive demand battery**.

	**Group**	**Baseline (0 h)**		**Sub-chronic (4 week)**		**ANCOVA**	
		***M***	***SD***	***M***	***SD***	***F***	***p***
**BEFORE CDB**							
Mental fatigue	Cocoa	51.05	14.83	54.53	16.07	0.70	0.41
	Placebo	47.89	14.05	49.76	14.42		
Stress	Cocoa	22.63	14.82	39.21	21.44	1.00	0.33
	Placebo	29.94	14.89	35.81	17.02		
**AFTER CDB**							
Mental fatigue	Cocoa	64.79	17.94	66.33	23.35	0.60	0.44
	Placebo	72.11	14.41	64.00	14.93		
Stress	Cocoa	36.95	19.14	40.56	22.64	5.50	0.03^*^
	Placebo	47.94	16.68	36.06	16.08		

*CDB, Cognitive Demand Battery*.

* *p < 0.05*.

During the first, second and third cycles of the CBD, there were no significant between-group differences for any of the tasks at the sub-chronic time point when co-varying for baseline data (Table 8).

**Table 8:** **Sub-chronic effects of cocoa supplementation on cognition: CDB cognitive tasks**.

		**Cycle 1**						**Cycle 2**						**Cycle 3**					
***CDB Task***	**Group**	**Baseline (0 h)**		**Sub-chronic (4 weeks)**		**ANCOVA**		**Baseline (0 h)**		**Sub-chronic (4 weeks)**		**ANCOVA**		**Baseline (0 h)**		**Sub-chronic (4 weeks)**		**ANCOVA**	
		***M***	***SD***	***M***	***SD***	***F***	***p***	***M***	***SD***	***M***	***SD***	***F***	***p***	***M***	***SD***	***M***	***SD***	***F***	***p***
Serial threes correct (number)	Cocoa	24.47	12.39	39.63	18.06	0.13	0.73	31.68	12.98	41.63	17.42	0.92	0.34	33.42	13.29	39.89	17.09	0.05	0.82
	Placebo	19.83	9.43	33.29	14.49			25.17	14.46	38.76	19.08			29.35	15.64	36.35	15.81		
Serial threes speed (ms)	Cocoa	5639.05	3426.91	3331.21	1617.37	0.28	0.60	3857.11	1876.65	3001.32	1408.70	0.04	0.84	3761.16	1697.78	3394.39	2560.07	0.68	0.42
	Placebo	6213.11	2487.61	3731.65	1329.49			4459.44	1546.01	3459.94	1697.89			4147.53	1521.50	3311.29	1222.84		
Serial sevens correct (number)	Cocoa	15.37	11.04	22.58	11.76	0.83	0.37	17.63	10.10	21.79	12.70	0.04	0.85	18.79	10.53	25.50	13.93	0.13	0.72
	Placebo	13.50	8.91	19.00	9.39			14.83	7.68	20.12	7.54			13.76	7.63	21.29	10.45		
Serial sevens speed (ms)	Cocoa	7561.05	4531.69	5659.89	2786.34	0.85	0.37	6603.95	3489.18	5062.21	2301.93	2.87	0.10	6472.42	3026.03	5578.11	3713.13	0.06	0.81
	Placebo	8304.89	3116.47	6749.76	3264.55			6733.94	1913.41	5828.00	2039.21			7235.47	2281.21	5914.18	2378.46		
RVIP accuracy (%)	Cocoa	44.97	24.02	51.97	24.53	0.51	0.48	49.54	24.81	50.79	26.34	0.08	0.78	43.51	30.32	45.56	27.37	0.13	0.72
	Placebo	32.64	15.13	46.62	13.32			40.00	17.09	43.10	18.76			38.08	18.26	45.74	21.41		
RVIP speed (ms)	Cocoa	452.78	58.71	462.40	45.67	1.64	0.21	472.21	57.02	469.86	60.76	1.41	0.24	475.10	40.25	471.05	53.15	0.72	0.40
	Placebo	470.89	54.87	488.69	44.62			493.91	49.29	501.40	51.32			493.75	53.52	491.83	45.30		

*RVIP, Rapid Visual Information Processing*.

#### Cardiovascular measures

No significant effects were found for any of the cardiovascular measures at the sub-chronic time point when co-varying for baseline data (Table 9).

**Table 9:** **Sub-chronic effects of cocoa supplementation on cardiovascular measures**.

	**Cocoa**				**Placebo**				**ANCOVA**	
	**Baseline (0 h)**		**Sub-chronic (4 week)**		**Baseline (0 h)**		**Sub-chronic (4 week)**			
	***M***	***SD***	***M***	***SD***	***M***	***SD***	***M***	***SD***	***F***	***p***
**CARDIOVASCULAR MEASURES**										
CCA velocity, cm/s	21.23	6.32	19.00	6.14	21.18	7.28	21.24	6.46	1.06	0.31
Heart rate, bpm	68.00	11.76	69.00	12.74	77.11	14.94	74.44	18.67	0.27	0.60
Systolic BP, mmHg	117.47	9.87	122.84	9.65	121.42	12.02	120.50	7.95	2.05	0.16
Diastolic BP, mmHg	70.68	9.42	71.84	8.15	71.21	9.04	72.61	7.73	0.21	0.65
Central systolic BP, mmHg	103.11	9.37	107.21	8.62	105.84	10.04	106.11	7.38	0.85	0.36
Central diastolic BP, mmHg	70.84	9.91	72.84	8.35	73.63	11.19	71.56	13.17	0.34	0.56
Mean arterial pressure, mmHg	84.53	10.16	87.05	9.20	88.00	11.71	88.33	9.10	0.02	0.90
Pulse pressure, mmHg	32.74	7.56	34.37	5.73	32.21	6.22	35.50	16.54	0.11	0.75
Augmented pressure, mmHg	2.89	3.48	3.28	4.28	2.52	2.56	3.18	4.00	0.001	0.97
Augmentation index, mmHg	8.50	11.70	8.89	11.49	8.29	7.98	9.41	10.43	0.03	0.86

*CCA, Common Carotid Artery; BP, Blood Pressure; mmHg, Millimeters of Mercury*.

## Discussion

This randomized, placebo-controlled, double-blind clinical trial investigated both the acute and sub-chronic effects of cocoa supplementation on cognitive performance. Consumption of 250 mg cocoa flavanols improved performance acutely on the Serial component of the Cognitive Demand Battery (CDB) in cycle one only. Furthermore, acute cocoa supplementation significantly reduced participants' self-reported mental fatigue prior to commencing the CDB testing battery. Following completion of the CDB at sub-chronic assessment, participants receiving the placebo supplement reported feeling significantly less stressed compared to participants supplemented with cocoa. No significant effects were found for cognition measured with the SUCCAB or cardiovascular function.

The acute improvements found in cognitive performance and mental fatigue are in line with the acute findings of Scholey et al. (2010). However, the present improvements in cognitive performance were found during the Serial Sevens component rather than for Serial Threes. Using a 520 mg cocoa beverage intervention, Scholey et al. (2010) found that participants produced more correct responses during the Serial Threes task throughout each of the six cycles (lasting 1 h, in comparison to the present study's three cycles lasting half an hour), with the most significant improvement seen during the fourth cycle at just over 2 h post cocoa consumption. Significant effects were also found for a 940 mg beverage; however the effect was weaker, producing significant improvements for the first four cycles only.

We found CDB effects only in the first cycle, whereas Scholey et al. (2010) found effects throughout six cycles. In the current study, participants did not begin the CDB until 3 h after taking the cocoa supplement, whereas participants in Scholey et al.'s (2010) study began the CDB one and a half hours post-consumption. Participants in our study completed the SUCCAB testing battery first, as a way of mentally taxing participants, before later completing the CDB. As the epicatechin component of cocoa flavanols peaks in concentration in human blood plasma at 2–3 h post consumption (Francis et al., 2006; Nehlig, 2013), it is possible that by completing the SUCCAB first, the CDB was pushed into a time frame where there were diminished levels of epicatechin. Additionally, as Scholey et al. (2010) used a much higher dose than was investigated in the current study, it is possible that participants in the current study had insufficient levels of epicatechin during the testing period.

At the acute time point, participants supplemented with cocoa reported feeling significantly less mentally fatigued prior to completing the CDB, than those receiving the placebo supplement. As the CDB was the final task in the testing sequence and was undertaken four and a half hours after the commencement of the experimental session, it is likely that participants may have been feeling fatigued at this point. It appears that at the time that mental fatigue was assessed, at 3 h post consumption, cocoa flavanols significantly attenuated this fatigue. While this effect was not carried through to the fatigue assessment after the CDB was completed, it is possible that this too relates to the fact that epicatechin levels were likely to have declined well past their peak effect by the end of the CDB (Francis et al., 2006; Nehlig, 2013),

Similar to results found by Crews et al. (2008), supplementation with cocoa flavanols did not produce any cognitive sub-chronic effects, with no significant changes found to performance on any of the CDB tasks or the SUCCAB test battery. Additionally, no significant changes to participants' self-reported mental fatigue were observed. The lack of sub-chronic effects on cognitive performance is also consistent with the findings of Pase et al.'s (2013). Interestingly, following completion of the CDB, participants that received placebo supplementation sub-chronically, reported feeling significantly less stressed than their cocoa counterparts. This result is unexpected, and in the absence of other sub-chronic effects, it may simply be a chance finding. It is noteworthy that participants had been instructed to abstain from the daily trial supplement on the morning of the sub-chronic testing (1 month follow up). In this way, any potential acute effects were eliminated. Therefore, our conclusions regarding the lack of sub-chronic effects are further bolstered by this aspect of the experimental design. However, this does not eliminate the possibility that there may be positive benefits with a longer cumulative supplementation period. Future studies should also consider the potential effects of acute, chronic and acute on chronic effects.

The exact mechanism behind the action of cocoa flavanols is yet to be conclusively determined. One study suggested that improvements in vascular health may partly underpin improvements in cognition following cocoa supplementation in adults who are in poor health (Desideri et al., 2012). However, in young healthy adults, increases in cerebral blood flow have been reported in the absence of significant behavioral effects (Francis et al., 2006). Although the present study did not find any effects on blood flow velocity through the common carotid artery using Doppler, this measure may be relatively insensitive, as compared to the more costly neuroimaging methods utilized by Francis et al. It is also possible that the 250 mg dose of cocoa flavanols used in this study was insufficient to influence cardiovascular and blood flow changes.

The present study had a number of limitations. Despite participants completing a practice run for each SUCCAB sub-test during testing sessions, there was not a separate testing day devoted to practice testing prior to the assessment of baseline performance for all tasks. As such, the possibility of practice effects occurring in this sample cannot be eliminated. Larger studies making use of a practice testing day are recommended to confirm the findings presented here. Additionally, the duration of this trial was only 30 days. The possibility that longer intervention periods may result in different effects cannot be ruled out. Future studies may also benefit from investigating the effects of cocoa supplementation using a higher dose, rather than the 250 mg dose used here. It is possible that the lack of cardiovascular effects, and subsequent lack of cognitive effects seen here may be a result of too low a dose of cocoa flavanols. A study by Brickman et al. (2014) for example, established a chronic, 3 month intervention using a diet containing either high (900 mg) or low (10 mg) cocoa flavanols in healthy, older participants aged 50–69 years. It was found that in the high flavanol diet group, both dentate gyrus function and cognitive performance on the ModBent task were enhanced. It is possible that these findings were a result of the higher dose used (900 mg compared to 250 mg in the present study). It should also be noted that the cocoa supplement used in the current study included 5.56 mg of caffeine, whereas the placebo did not. Although the effects of such a small dose of caffeine on cognition are unclear, future research could match the placebo for caffeine content. Furthermore, the young age and size of the present cohort may also have contributed to our lack of cognitive and vascular findings.

In conclusion, a 250 mg dose of cocoa flavanols was found to attenuate mental fatigue and improve minor aspects of cognitive performance acutely but not sub-chronically during a highly demanding task. Future research should assess a higher dose of cocoa as well as cognitive, mood and cardiovascular effects in an older cohort.

### Conflict of interest statement

Sponsors were not involved in study design, data collection, analysis, interpretation and writing of the article. Swisse Wellness Pty Ltd. provided cocoa and placebo tablets for the study, however there was no commercial interest in the study. Any affiliations of authors with sponsors of the study are independent of the research project. All authors declare no conflicts of interest. The authors declare that the research was conducted in the absence of any commercial or financial relationships that could be construed as a potential conflict of interest.
